# Detection of *Bartonella bovis* DNA in blood samples from a veterinarian in Mexico

**DOI:** 10.1590/S1678-9946202365062

**Published:** 2023-12-04

**Authors:** Jannete Gamboa-Prieto, Anabel Cruz-Romero, José A. Jiménez-Hernández, José Rodrigo Ramos-Vázquez, Gerardo G. Ballados-González, Dora Romero-Salas, Violeta T. Pardío-Sedas, Sandra C. Esparza-Gonzalez, Ingeborg Becker, Sokani Sánchez-Montes

**Affiliations:** 1 Universidad Veracruzana, Facultad de Medicina Veterinaria y Zootecnia, Laboratorio de Enfermedades Infecciosas, Veracruz, Mexico; 2 Universidad Veracruzana, Facultad de Medicina Veterinaria y Zootecnia, Laboratorio de Parasitología, Veracruz, Mexico; 3 Universidad Veracruzana, Facultad de Medicina Veterinaria y Zootecnia, Laboratorio de Seguridad Agroalimentaria, Veracruz, Mexico; 4 Universidad Autónoma de Coahuila, Facultad de Odontología, Coahuila, Mexico; 5 Universidad Nacional Autónoma de México, Facultad de Medicina, División de Investigación, Centro de Medicina Tropical, Ciudad de México, Mexico; 6 Universidad Veracruzana, Facultad de Ciencias Biológicas y Agropecuarias, región Tuxpan, Tuxpan de Rodríguez Cano, Veracruz, Mexico

**Keywords:** Bartonellosis, Occupational risk, Zoonoses, Veterinary health professionals

## Abstract

The genus *Bartonella* encompasses 38 validated species of Gram-negative, facultative intracellular bacteria that colonize the endothelial cells and erythrocytes of a wide spectrum of mammals. To date, 12 *Bartonella* species have been recorded infecting humans, causing diseases of long historical characterization, such as cat scratch fever and trench fever, and emerging bartonellosis that mainly affect animal health professionals. For this reason, this study aimed to report a documented case of *Bartonella bovis* infecting a veterinarian from Mexico by the amplification, sequencing and phylogenetic reconstruction of the citrate synthase (*gltA*) and the RNA polymerase beta-subunit (*rpoB*) genes, and to report the natural course of this infection. To our knowledge, this work is the first to report the transmission of *B. bovis* via needlestick transmission to animal health workers in Latin America.

## INTRODUCTION

The genus *Bartonella* encompasses a megadiverse group of Gram-negative, facultative intracellular bacteria that colonize the endothelial cells and erythrocytes of a wide spectrum of mammals. In this host group, these bacteria can generate long-lasting bacteraemia with multiple clinical signs, among which haematological, hepatic, and neurological manifestations stand out, varying in severity depending on the infecting *Bartonella* species and the host’s immunological status^1–3^. Various arthropod species—most importantly fleas, lice, sandflies, and mites—are involved in the transmission of these bacteria^3,4^. To the best of our knowledge, 38 *Bartonella* validated species have been identified globally, of which at least 12 have been confirmed to be zoonotic^4,5^. The *Bartonella* bacteria most commonly found in humans are *Bartonella bacilliformis, Bartonella quintana*, and *Bartonella henselae*; however, the zoonotic potential of many other species is as yet unknown^4,6^.

Animal health professionals are a risk group for zoonoses, since they are exposed to a wide range of potentially *Bartonella*-infected vertebrate hosts and their blood-sucking arthropods^7^. Veterinarians are constantly at risk of acquiring species of these bacteria through bites, scratches and/or contact with the fluids of infected animals, as well as through the iatrogenic route^7–10^. A certain group of *Bartonella* species is associated with artiodactyls (two toes) and perissodactyls (one or three toes), which have increasingly attracted the interest of public health professionals in recent years^11^. This group includes the *Bartonella chomelii* and *Bartonella bovis*, which have been detected in cattle from Algeria, Lithuania, and the USA, generating persistent infections in their hosts^11–14^. There is also a documented report of *B. bovis* causing endocarditis in a pregnant Angus cow in the USA^14^. Animal production has increased due to the growing food demand worldwide, which has intensified concerns around the fact that veterinarians who work with livestock may be exposed to *Bartonella* species associated with these animals, causing diseases that could be underdiagnosed. For this reason, this study aimed to report the first documented case of *B. bovis* infecting a veterinarian from Mexico and to report the natural course of this infection.

### Ethical statement

All procedures were carried out in accordance with the ethical standards of the institution or practice at which the studies were conducted. The current report was approved by the Research Ethics Committee of the Medical Faculty of the Universidad Nacional Autonoma de Mexico, FMED/CI/JMO/129/2017). The patient signed a written consent form for the publication of the case.

## CASE REPORT

**Figure 1:**
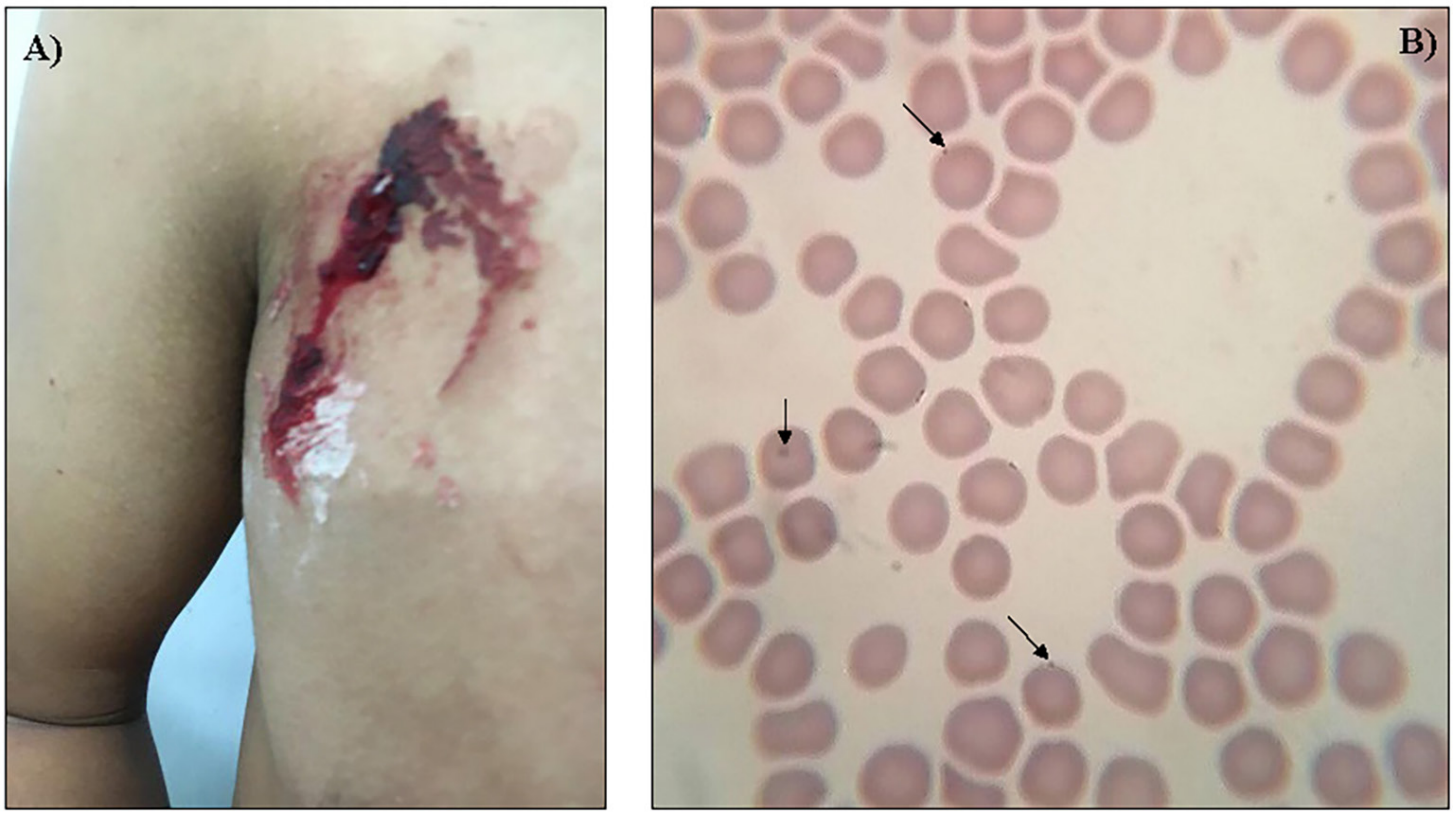
Identification of *Bartonella bovis* DNA in a veterinarian: A) Photograph of the chest lesion taken immediately after the needlestick; B) Identification of intracellular inclusions in erythrocytes from the patient’s sample.

**Figure 2:**
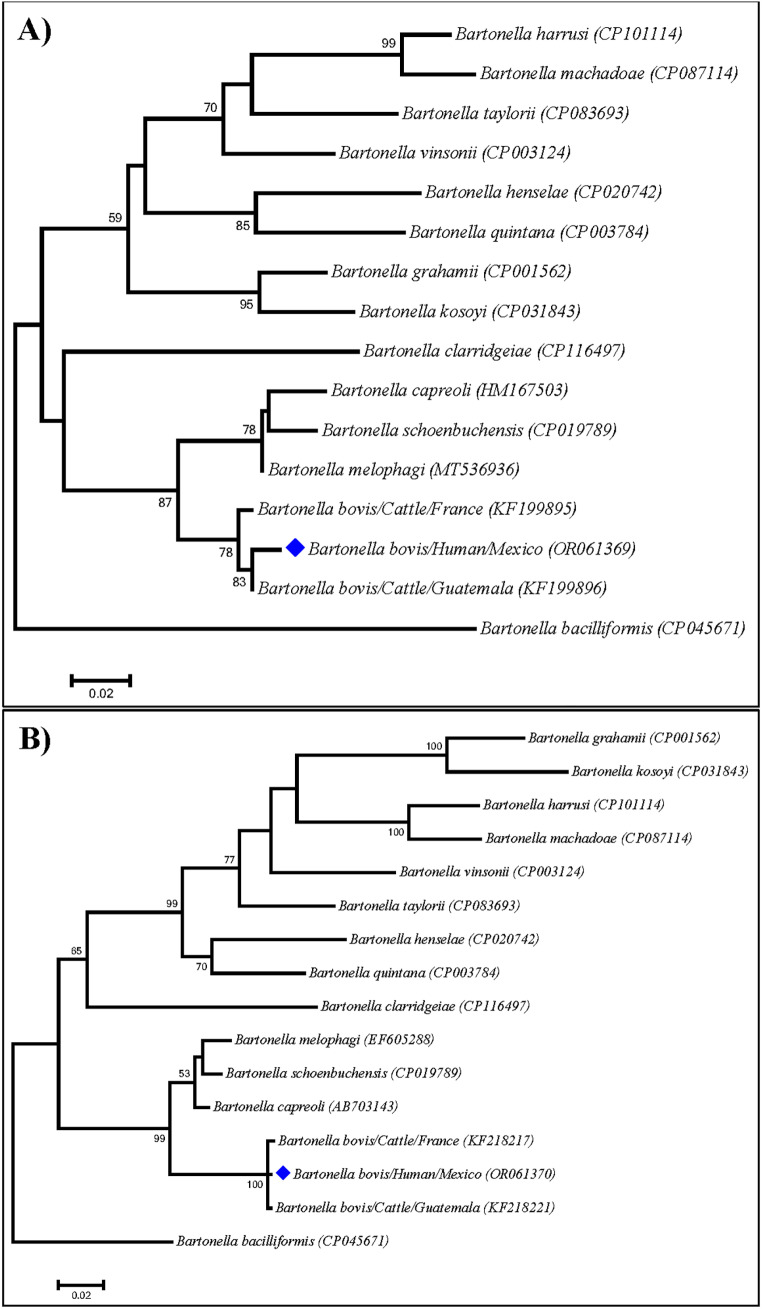
Maximum likelihood phylogenetic trees generated with partial sequences of the *gltA* (A) and *rpoB* (B) genes from several members of the genus *Bartonella* using the General Time Reversible (GTR) distance model with gamma distribution (+G). Blue diamonds indicate sequences generated in Mexico, Bootstrap values higher than 50 are indicated in the nodes.

On 14 June 2020, a 35-year-old male veterinarian working at a cattle ranch in the town of Ocotillo, in the municipality of Colipa, Veracruz, Mexico, accidentally inoculated his right pectoral with an implant needle while implanting lactating calves. Immediately afterwards, he washed the wound with soap and water (Figure 1A) and resumed his activities, deworming, vaccinating and applying implants in cattle of different ages. Upon returning to his home, he once again cleaned the area and applied an antiseptic (sodium hypochlorite, hypochlorous acid, and free chlorine) to the it. Thirteen days after inoculation, the patient began to manifest symptoms such as headache, intermittent fever (36.2 °C–39.0 °C), chills, sweating, joint and retro-ocular pain, severe fatigue, and tachycardia. For this reason, he decided to go to the hospital, where he was told to undergo laboratory studies to rule out viral diseases such as COVID-19 (through PCR and immunochromatography [both came back negative]) and dengue (through the detection of IgM and IgG antibodies, and through immunochromatographic testing [negative]), and bacterial diseases (Brucellosis and Salmonellosis [through febrile reactions]). The patient also underwent laboratory studies: serial blood counts and C-reactive protein (Table 1). His heart rate (70–80 bpm), oxygen saturation (98%), and body temperature were also normal after August 1^st^ and were kept that way for the subsequent seven days. The patient self-medicated with 500 mg paracetamol every six hours; zinc; vitamins A, D and C; acetylsalicylic acid; and oral hydration with electrolytes, but his organism did not respond favorably to this type of treatment. All tests for the detection of infectious agents had come back negative up to this point. From 27 and 28 June onward, the patient experienced leukopenia, thrombocytopenia, lymphocytosis and alterations in C-reactive protein and D-dimer. Professionals decided to perform a Giemsa-stained blood smear for the microscopic identification of hemoparasites, and could visualize inclusions in erythrocytes compatible with members of the genera *Anaplasma, Bartonella*, and *Mycoplasma* (Figure 1B). To allow for a differential diagnosis of the infective bacterial group, a blood sample was sent to the Center for Tropical Medicine (CMT) of the National Autonomous University of Mexico (UNAM), where professionals would perform polymerase chain reaction tests for the detection of DNA of any of the three previously referred agents. For this, the total genomic DNA was extracted using the Qiagen DNEasy Blood and Tissue Kit, following the supplier’s specifications. For the molecular detection of the three agents, fragments of the *16S-rDNA* gene for *Anaplasma* and *Mycoplasma*, as well as the citrate synthase (*gltA*) and the RNA polymerase beta-subunit (*rpoB*) genes for *Bartonella*, were amplified and sequenced using primers and thermal conditions previously reported by Martínez-Hernández *et al*.^15^ and Lozano-Sardaneta *et al*.^16^. PCR products were evaluated on 2% agarose gels stained with SmartGlow. Positive amplicons were sent to Macrogen, Korea for sequencing. The recovered sequences were compared with those of other valid pathogen species using the BLASTn online tool and subsequently used to generate global alignments using the CLUSTAL W paired algorithm. Lastly, a phylogenetic reconstruction was conducted in Mega 11.0 using the Maximum Likelihood method, with 10,000 Bootstrap iterations and removing the gaps. Molecular assays did not detect *Anaplasma* or *Mycoplasma* DNA; but the blood sample tested positive for *Bartonella*. Using BLAST analysis, these sequences were found to be 99% (353/356 bp) and 100% (779/780 bp) identical to the corresponding sequences of the *gltA* (MN615935, and KF199896) and *rpoB* (KF218218) genes, respectively, of *B. bovis* from Brazil and Guatemala. A Maximum Likelihood analysis confirmed that the bacteria collected from the patient corresponded to *B. bovis*, because it grouped with other *B. bovis* sequences detected in cattle from Guatemala and France, with a support value of 100 (Figure 2). Sequences generated in this study were deposited in GenBank under Accession numbers OR061369 and OR061370. Once the presence of *Bartonella* DNA was confirmed in the patient’s blood sample, he began specific treatment with azithromycin (500 mg) every 24 hours, for 15 days. A PCR was performed five days after the patient completed his treatment, as well as one month later. Since both tests came back negative and no evidence of symptomatology was found, the patient was discharged.

**Table 1:** Laboratory test results of a patient infected by *Bartonella bovis*, Veracruz, Mexico, 2020.

DATE	Hematologic parameter													Febrile reaction test							Blood chemistry parameter		
	HEM x 10^6^/mm^3^	Hbgr/dL	HCT%	VGMfL	HCMpg	PLTx 10^3^/mm^3^	LEUx 10^3^/uL (%)	NEU/uL (%)	EOS/uL (%)	BAS/uL (%)	MON/uL (%)	LINF/uL (%)		Paratiphic A	Paratiphic B	Tiphic -H	Tiphic-O	*Proteus* Ox-19	*Brucella abortus*		C-reactive protein mg/L	LDH U/L	D dimer ng/mL
Reference values	3.8-5.8	11.0-16.5	35.0-50.0	80.0-97.0	32.0-34.5	150-450	5000-10000	1500-6600	0-700	0-300	0-800	1500-3500		Negative	Negative	Negative	Negative	Negative	Negative		<6	240-480	0-386
June 27^th^	4.5	13.7	40.9	90.9	33.5	**136**	**4720 (4.72)**	2974 (63)	0 (0)	0 (0)	189 (4)	**1086 (23)**		NR	NR	NR	NR	NR	NR		NR	NR	NR
June 28^th^	4.4	13.6	41	41.5	30.4	**115**	**3290 (3.29)**	**1481 (45)**	33 (1)	33 (1)	263 (8)	**1283 (39)**		Negative	Negative	Negative	Negative	Negative	Negative		**21.1**	279	**447**
June 29^th^	4.6	14.2	42.9	92.5	30.6	**133**	**3380 (3.38)**	**1453 (43)**	169 (5)	0 (0)	304 (9)	**1318 (39)**		NR	NR	NR	NR	NR	NR		NR	NR	NR
July 6^th^	5.1	15.3	47.2	90.9	29.5	225	**4060 (4.06)**	2395 (59)	122 (3)	0 (0)	284 (7)	**1218 (30)**		NR	NR	NR	NR	NR	NR		NR	NR	NR
August 3^rd^	4.8	14.5	44.3	91.2	32.7	177	**4750 (4.57)**	2011 (44)	137 (3)	46 (1)	229 (5)	1965 (43)		NR	NR	NR	NR	NR	NR		NR	NR	NR

HEM = erythrocytes; Hb = hemoglobin; HCT = hematocrit; VGM = mean erythrocyte volume; HCM = mean corpuscular hemoglobin; PLT = platelets; LEU = leukocytes; NEU = neutrophils; EOS = eosinophils; BAS = basophils; MON = monocytes; LINF = lymphocytes; LDH = lactic dehydrogenase; NR = no results. Values in bold are out of range.

## DISCUSSION

The number of reports of animal health professionals infected with various species of the genus *Bartonella* has increased exponentially in recent years^8–10^. The frequency and occurrence of infections by species such as *B. henselae* have increased in veterinary clinics for small companion animals and in large production animal veterinary clinics in Spain and the United States^7–9^. Additionally, there is a report of a suspected transmission, via needlestick, of *Bartonella vinsonii* subspecies *berkhoffii* to a veterinarian through accidental autoinoculation with infected blood from a dog^17^. Given this background and the findings of this study, health professionals must be taught to be aware of the risk of needle stick transmission when handling patients, of the importance of wearing personal protective equipment, and of the immediate attention needed to deal with incidents involving sharp surgical material, as well as the need for post-incident surveillance for signs consistent with bartonellosis.

Despite the factors mentioned and the severity of this problem, there are no studies assessing contact or direct work with large species, particularly cattle, as a risk factor of infections by pathogens prevalent in livestock. In this study, sequencing and phylogenetic analysis confirmed that *B. bovis* infected a veterinarian after a work accident due to inoculation with bovine blood. *Bartonella bovis* is a microorganism that was first detected in dual-purpose cattle in Europe, in 2002^18^. This species has also been found in countries such as Guatemala and the US, causing lesions consistent with endocarditis in adult cattle^19^.

## CONCLUSION

This case is a priority because it demonstrates the infective capacity of *B. bovis* in humans and calls attention to the risk of exposure in certain occupations. *Bartonella bovis* was recently reported in Mexico, specifically in productive cattle farms in central Veracruz^20^. This work demonstrates the need to consider this species a causal agent of febrile illness in animal health professionals, animal handlers, slaughterhouse workers, and by-product handlers.
